# Fractional topological phase measurement with a hyperentangled photon source

**DOI:** 10.1038/s41598-018-37344-6

**Published:** 2019-01-24

**Authors:** A. A. Matoso, R. A. Ribeiro, L. E. Oxman, A. Z. Khoury, S. Pádua

**Affiliations:** 1 Departamento de Física, Universidade Federal de Minas Gerais, 31270-901 Belo Horizonte, Minas Gerais Brazil; 2 Institut für Angewandte Physik, Universität Bonn, Wegelerstr. 8, 53115 Bonn, Germany; 3 Instituto de Física, Universidade Federal Fluminense, 24210-346 Niterói, Rio de Janeiro Brazil

## Abstract

Pairs of photons simultaneously entangled in their path and polarization degrees of freedom are used to measure the topological phase acquired by bipartite entangled states. Conditional phase local unitary operations having the polarization degree of freedom as the control variable are applied. Qudits of arbitrary dimensions are encoded on the photons transverse positions while polarization entanglement is used as an auxiliary resource for quantum interference measurements. With this scheme the fractional phases predicted for dimensions *d* = 2, 3 and 4 could be measured with visibilities for the interference curves beyond the limit allowed for classical sources, which is expected for a source of quantum correlated photons. The strategy of perform a quantum interferometry experiment with photons entangled in an auxiliary degree of freedom and apply unitary local operations conditioned to this auxiliary variable shows an increase to the signal to noise ratio, simplifies alignment and can be used in different applications. This offers an interesting perspective for the efficient implementation of phase gates in quantum computing with hyperentangled photon sources in polarization and path degrees of freedom. Furthermore, one can conjecture whether the measured phase can serve as a dimensionality identifier of the Hilbert space dimension for an unknown state preparation.

## Introduction

Topological phase investigation was preceded historically by geometrical phase. The latter emerged in the context of quantum mechanics in 1984 with Berry^1^, who investigated the evolution of a quantum state under the action of a time dependent Hamiltonian. He considered that the time dependency (also present in the eigenstates) occurs through a set of parameters modified according to the adiabatic approximation. Therefore, the geometric interpretation was in charge of the parameter space. Three years later, Aharonov and Anandan^2^ generalized the geometrical phase definition without the need to consider the parametrization and the adiabatic approximation. Thus, the geometric interpretation was transferred to the so called projective Hilbert space, which is the set obtained when all pure states that differ by a multiplicative phase factor are identified as a single element. For example, the projective Hilbert space for qubits is mapped on the Bloch sphere.

In 1991, Kwiat and Chiao^3^ observed the geometrical phase at single photon level using bipartite photon systems for its detection. This work was followed by Martienssen *et al*.^4^ and Shih and Strekalov^5^. However, in these works the goal was still to observe the geometrical phase acquired by a quantum state when it evolves according to a path in the projective Hilbert space. Sjöqvist was the first to point out the connection between entanglement and discrete geometrical phases in 2000^6,7^. He found that maximally entangled two-qubit states can only acquire geometrical phases equal to 0 or *π*. Later, Milman and Mosseri^8,9^ discussed their topological aspects through the double connectedness of the *SO*(3) group. In refs^10–12^ the authors make use of the term “topological” in a different meaning from the one discussed here. There, “topological” means that the geometrical phase depends only on the path followed in the projective Hilbert space, while in this article fractional topological phases are related to the double or multiple connectedness of the projective space.

The first experimental demonstrations of discrete phases for qubits were achieved by means of interferometric techniques with spin-orbit laser modes^13^ and with conditional operations on an ancilla qubit in Nuclear Magnetic Resonance^14^. The discrete phases were later extended to qudits of arbitrary dimension *d* by Oxman and Khoury, when fractional phase values, in multiples of 2*π*/*d*, were predicted^15^. Different works appeared since then, defining the topological phase for multiqubit systems^16^, proposing experiments for photonic qudits^17^ and multiqubits^18^, generalizing the theory for two qudits with different dimensions^19^, and investigating the entanglement dependency for two qubits^20^. Experimental works have been devoted to measuring fractional topological phases in two-photon interference^21^. However, the visibilities obtained were below the limit allowed by classical correlations and the quantum nature of the interference fringes could not be ascertained.

In this work we employ a hyperentangled photon source to demonstrate two-qudit fractional topological phases with visibilities beyond the limit allowed by classically correlated sources. Pairs of photons simultaneously entangled in their polarizations and transverse positions are generated by spontaneous parametric down conversion. Our source is composed by two non linear crystals cut for type I phase match, with their optical axes orthogonally oriented. The photonic qudits are encoded in the transverse positions of the pair, while polarization is used as a resource for measuring the topological phase without the need of longitudinal path interference. This scheme allows the local *SU*(*d*) transformations to be simultaneously applied to both photons of the pair. Cyclic evolutions are accomplished with independent operations on the qudits, thus stressing the nonlocal nature of the fractional topological phases. The production of hyperentangled photons allows direct measurement of the topological phase without longitudinal path interferometry or post selection procedures. This provides a more reliable demonstration of the fractional phase once the fringe visibilities are guaranteed to be above the limit allowed by classically correlated sources and the simultaneous operation of both qudits is now possible. These are important advancements brought by the present scheme as compared to the previous one published in ref.^21^.

## Fractional Topological Phases for ***d*** × ***d*** Bipartite Pure States

Let us consider a two-qudit system initially prepared at time *t* = 0 in an arbitrary pure state $$|\phi (t=0)\rangle =$$$${\sum }_{mn}\,{\alpha }_{mn}(t=0)|mn\rangle $$ (1 ≤ *m*, *n* ≤ *d*). The fractional topological phases are naturally realized in the polar decomposition of the coefficient matrix^15,19,22^

1 $$\alpha (0)={e}^{i\varphi (0)}Q(0)S(0),$$

where *Q*(0) is a Hermitian matrix, *S*(0) is an SU(*d*) matrix and *ϕ*(0) is an overall phase factor. Under local unitary operations $U(t)={e}^{i{\varphi }_{s}(t)}\bar{U}(t)$ and $V(t)={e}^{i{\varphi }_{i}(t)}\bar{V}(t)$, with $\bar{U}$ and $\bar{V}$ being SU(*d*) matrices, the polar decomposition evolves in time. This means that it holds during the state evolution for any instant *t*, and it is possible to write explicitly how the evolution occurs for *ϕ*(*t*), *Q*(*t*) and *S*(*t*), the sectors of the polar decomposition. The evolved coefficient matrix *α*(*t*) becomes

2 $$\alpha (t)=U\alpha (0){V}^{T}={e}^{i\varphi (t)}Q(t)S(t),$$

where *ϕ*(*t*) = *ϕ*_*s*_(*t*) + *ϕ*_*i*_(*t*) + *ϕ*(0), $Q(t)=\bar{U}Q(0){\bar{U}}^{\dagger }$ and $S(t)=\bar{U}S(0){\bar{V}}^{{\rm{T}}}$. Here the subscripts *s* and *i* in the phases *ϕ* anticipate the connection with the photons signal and idler in the experiment. The phase acquired by the two-qudit quantum state is computed from the overlap $\langle \phi (0)|\phi (t)\rangle ={\rm{Tr}}\,[{\alpha }^{\dagger }(0)\alpha (t)]$, between the evolved and the initial states. The fractional phases arise naturally in the SU(*d*) sector when we consider a cyclic evolution *α*(0) → *α*(*t*) = *e*^*i*Φ^*α*(0). The phase change can be inspected in each sector of the coefficient matrix. First, we have a trivial contribution from the U(1) sector associated to the overall phase factor *ϕ*(*t*). Then, Hermiticity prevents any phase contribution from *Q*(*t*). Finally, the condition det *S*(*t*) = 1, defining the SU(*d*) matrices, restricts any phase factor from this sector to *d*-th roots of the unity. Therefore, any phase contribution from the SU(*d*) sector in a cyclic evolution must be of the fractional form *e*^2*inπ*/*d*^ ($n\in {\mathbb{Z}}$), giving rise to fractional phases

3 $${\gamma }_{top}=\frac{2n\pi }{d}.$$

Moreover, when the qudits are operated by local SU(*d*) transformations, the U(1) sector remains stationary and the fractional phases are the only ones attainable under cyclic evolutions. These fractional phases are from topological nature since the set $\{S\in {\rm{SU}}(d)|{e}^{i\gamma }S\equiv S\}$ is not simply connected and different homotopy classes of closed trajectories can be conceived. A detailed analysis for qubits can be found in refs^8,9^.

## Measurement Strategy

Quantum interference is the natural way to evince the fractional phases produced by local unitary transformations on qudit pairs. These phases can be measured by interfering the evolved state with a copy of the initial state, what requires an auxiliary degree of freedom to distinguish the two copies and to allow projective operations leading to the superposition between the final and initial two-qudit states. In this context, entanglement in both degrees of freedom (hyperentanglement) will prove useful.

Let us consider a photon pair (signal and idler) generated by spontaneous parametric down conversion (SPDC), simultaneously entangled in their polarization and transverse positions. The two-photon polarization state produced by the SPDC source can be prepared as

4 $$\frac{|hh\rangle +|vv\rangle }{\sqrt{2}}.$$

Each photon is sent through a mask carrying *d* slits that encode a spatial qudit in each photon’s transverse position. The two-qudit state generated by the masks is

$$|\phi (0)\rangle =\sum _{m,n=1}^{d}\,{\alpha }_{mn}(0)|mn\rangle .$$

Therefore, the complete quantum state of the photon pair is

5 $$\begin{array}{rcl}{\rm{\Psi }} & = & {(\frac{|hh\rangle +|vv\rangle }{\sqrt{2}})}_{pol}\otimes {|\phi (0)\rangle }_{tp}\\  & = & \sum _{m,n=1}^{d}\,\frac{{\alpha }_{mn}(0)}{\sqrt{2}}({|m,h\rangle }_{s}{|n,h\rangle }_{i}+{|m,v\rangle }_{s}{|n,v\rangle }_{i}).\end{array}$$

A conditional gate is then applied to the transverse position of each photon, controlled by their polarization state. This realizes the following transformations

6 $$\begin{array}{c}|\phi (0)\rangle \otimes |hh\rangle \to |\phi (t)\rangle \otimes |hh\rangle ,\\ |\phi (0)\rangle \otimes |vv\rangle \to |\phi (0)\rangle \otimes |vv\rangle .\end{array}$$

In the experiment, this is achieved by means of a spatial light modulator (SLM). The SLM performs local unitary transformations $U\otimes V$ in the spatial qudits^21^, producing

7 $$\begin{array}{rcl}|\phi (t)\rangle  & = & U(t)\otimes V(t)|\phi (0)\rangle \\  & = & \sum _{m,n=1}^{d}\,{\alpha }_{mn}(t)|mn\rangle .\end{array}$$

After the conditional gate, the two-photon state becomes

8 $$|{\rm{\Psi }}^{\prime} \rangle =\frac{|\phi (t)\rangle |hh\rangle +|\phi (0)\rangle |vv\rangle }{\sqrt{2}},$$

where it is clear the preparation of a superposition state between the two qudit state at *t* = 0 (tensor to $|hh\rangle$ state) and the evolved state at a general instant *t* (tensor to $|vv\rangle$ state). This state superposition state in the path variables is necessary for measuring the fractional topological phase. The advantage of this experimental scheme as compared with the interferometer of ref.^21^ is that here we do not need to post select this superposition state from a more general state. Our technique already prepares the necessary state for measuring the topological phase.

Interference between the transformed and the initial qudit states can be achieved by introducing a relative phase between signal and idler arms, followed by a projection in the two-photon polarization. In our scheme, a phase shifter is used to make $|h\rangle \to {e}^{-2i\theta }|v\rangle$ and $|v\rangle \to {e}^{2i\theta }|h\rangle$ on one of the twin photons. Then, a half-wave plate (HWP) and a polarizing beam splitter (PBS) are placed on each photon’s path. The HWPs are oriented to make $|hv\rangle \to |+-\rangle$ and $|vh\rangle \to |-+\rangle$, giving

9 $$|{\rm{\Psi }}^{\prime\prime} \rangle =\frac{{e}^{-2i\theta }|\phi (t)\rangle |+-\rangle +{e}^{2i\theta }|\phi (0)\rangle |-+\rangle }{\sqrt{2}},$$

where $$|\pm \rangle =(|h\rangle \pm |v\rangle )/\sqrt{2}$$.

An avalanche photodetector (APD) is placed after each PBS and the normalized two-photon coincidence count is proportional to the normally ordered intensity correlation function

10 $$C=\int \,{\Vert {E}_{h}^{+}({{\bf{r}}}_{{\bf{i}}}){E}_{h}^{+}({{\bf{r}}}_{{\bf{s}}})|{\rm{\Psi }}^{\prime\prime} \rangle \Vert }^{2}\,{{\bf{d}}}^{{\bf{2}}}{{\bf{r}}}_{{\bf{i}}}\,{{\bf{d}}}^{{\bf{2}}}{{\bf{r}}}_{{\bf{s}}},$$

where $${E}_{h}^{+}({{\bf{r}}}_{{\bf{j}}})={\hat{e}}_{h}\cdot {{\bf{E}}}^{+}({{\bf{r}}}_{{\bf{j}}})$$ and

11 $${{\bf{E}}}^{+}({{\bf{r}}}_{{\bf{j}}})=\sum _{m=1}^{d}\,({a}_{hm}^{j}{\hat{{\bf{e}}}}_{h}+{a}_{vm}^{j}{\hat{{\bf{e}}}}_{v}){f}_{m}({{\bf{r}}}_{{\bf{j}}}),$$

with *j* = *s*, *i* is the positive frequency component of the electric field operator, given in terms of the orthonormal slit mode functions *f*_*m*_(***r***_***j***_), the polarization unit vectors ${\hat{{\bf{e}}}}_{h}$ and ${\hat{{\bf{e}}}}_{v}$, and the associated annihilation operators ${a}_{hm}^{j}$ and ${a}_{vm}^{j}$. The expression given in Eq. (10) can be easily worked out to give

12 $$C(\theta )=1+v\,\cos (4\theta -\delta ),$$

where $v=|\langle \phi (0)|\phi (t)\rangle |$ and $\delta ={\rm{\arg }}\,\langle \phi (0)|\phi (t)\rangle$. In our measurements, the SU(*d*) operations are parametrized by $t\in [0,1]$. For fixed *t*, the phase shift *θ* will be varied to provide interference fringes. Then, the parameter *t* can be tuned resulting in the appearance of the fractional phases as discrete displacements of the fringes when the visibility *v* is maximized, since in this situation we have a cyclic evolution as in the discussion following Eq. (2). Starting from $\bar{U}(0)=\bar{V}(0)=1$, the fringe visibility is maximized whenever a cyclic operation is completed.

In order to prevent extra dynamical phases, the local unitary operations applied to the spatial qudits will be restricted to SU(*d*) and the SLM will be programmed to perform $\bar{U}={\rm{diag}}[{e}^{i{\xi }_{1}},{e}^{i{\xi }_{2}},\ldots ,{e}^{i{\xi }_{d}}]$ and $\bar{V}={\rm{diag}}[{e}^{i{\chi }_{1}},{e}^{i{\chi }_{2}},\ldots ,{e}^{i{\chi }_{d}}]$, with ${\sum }_{m=1}^{d}\,{\xi }_{m}={\sum }_{n=1}^{d}\,{\chi }_{n}=0$. If ${\sum }_{m}\,{\xi }_{m}=c$ for example, we can put the factor *e*^*ic*/*d*^ in evidence in the matrix and redefine each phase as ${\xi ^{\prime} }_{m}=\xi -c/d$, and now ${\sum }_{m}\,{\xi ^{\prime} }_{m}=0$. Therefore, ${\sum }_{m}\,{\xi }_{m}\ne 0$ only results in a extra dynamical phase that can be incorporated in *ϕ*_*s*_. Then, the overlap between the initial and the evolved quantum states becomes

13 $$\langle \phi (0)|\phi (t)\rangle =\sum _{m,n=1}^{d}\,|{\alpha }_{mn}(0){|}^{2}\,{e}^{i[{\xi }_{m}(t)+{\chi }_{n}(t)]}.$$

From expressions (12) and (13) we can calculate the expected interference curve once the initial state and the SU(*d*) operations have been specified. We will focus on maximally entangled two-qudit states that can be generated in the SPDC. The condition of transverse momentum conservation in this phenomenon naturally leads to the maximally entangled state (MES) with anti-correlations when the pump angular spectrum is properly manipulated^23^,

14 $$|\phi (0)\rangle =\frac{1}{\sqrt{d}}\,\sum _{m=1}^{d}\,|m,d-m+1\rangle ,$$

characterized by ${\alpha }_{mn}={\delta }_{m,d-n+1}/\sqrt{d}$. One could inquire how Eq. (12) would be affected by both polarization and path partial entanglement. If the coefficients module in Eq. (8) are different from $1/\sqrt{2}$, this would only change the interference visibility. The same happens if the coefficients |*α*_*mn*_|^2^ in Eq. (13) are different from *δ*_*m*,*d*−*n*+1_/*d*. Therefore, despite of the fact that partial entanglement does not affect the phase displacement, we must be careful when generating the hyperentangled state in order to guarantee the quantum nature of the two-photon interference.

The following local SU(*d*) operations will be performed on qudit pairs with *d* = 2, 3, 4:

- *d* = 2
  15 $$\begin{array}{c}{\xi }_{1}={\chi }_{2}=\frac{\pi }{2}t\\ {\xi }_{2}={\chi }_{1}=-\,\frac{\pi }{2}t\end{array}$$
- *d* = 3
  16 $$\begin{array}{c}{\xi }_{1}={\chi }_{3}=\frac{2\pi }{3}t-\frac{\pi }{3}(2t-1)H(t-\frac{1}{2})\\ {\xi }_{2}={\chi }_{2}=-\,\frac{2\pi }{3}t\\ {\xi }_{3}={\chi }_{1}=\frac{\pi }{3}(2t-1)H(t-\frac{1}{2})\end{array}$$
- *d* = 4

17 $$\begin{array}{c}{\xi }_{1}={\chi }_{4}=-\,\frac{\pi }{4}t+\frac{\pi }{2}(1-2t)H(t-\frac{1}{2})\\ {\xi }_{2}={\chi }_{3}=\frac{\pi }{4}t\\ {\xi }_{3}={\chi }_{2}=\frac{3\pi }{4}t-\frac{\pi }{2}(1-2t)H(t-\frac{1}{2})\\ {\xi }_{4}={\chi }_{1}=-\,\frac{3\pi }{4}t\end{array}$$

where *H*(*t*) is the Heaviside function. The expected curves are shown in the first row of the comparative panel in section “Experimental Results” for three values of *t*. In all cases the fringe visibilities vanish when *t* = 0.5 and recovers its maximum at *t* = 1 with the curve shifted by 2*π*/*d*.

## Interference Measurements on Hyperentangled Photon Pairs

**Figure 1:**
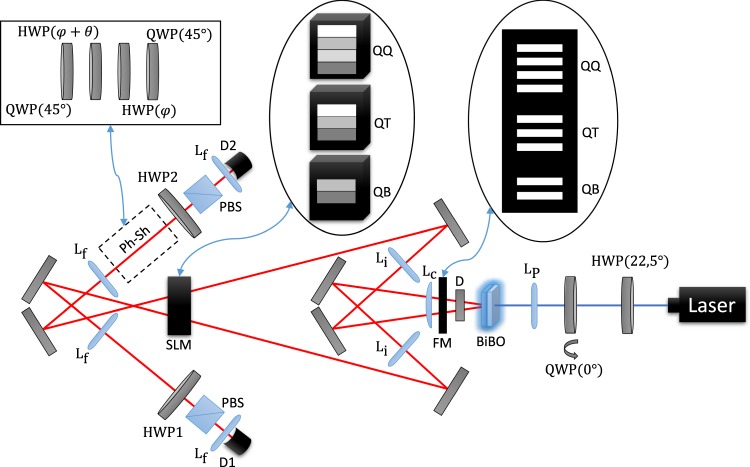
Experimental setup for measuring fractional topological phases. HWP: half-wave plate, QWP: quarter-wave plate, *L*_*p*_: spherical lens with 30 cm focal length, D: dichroic mirror, MS: multiple-slit, *L*_*c*_: cylindrical lens, *L*_*i*_ spherical lens with 15 cm focal length, Ph-Sh: phase shifter composed by a QWP at 45°, a HWP at *θ*, another HWP at *θ* + *ϕ*, and another QWP at 45°. *L*_*f*_: spherical lens, D1 and D2: detectors, PBS: Polarizing beam splitter.

The scheme of the experimental setup is shown in Fig. 1.

A 355 nm pump laser beam passes through a half-wave plate oriented at 22.5° which leaves the light beam linearly polarized at 45°. Then a quarter-wave plate slightly tilted with respect to the pump direction introduces a relative phase *e*^*iλ*^ between the *h* and *v* components of the pump beam. After passing through *L*_*P*_, the laser pumps two BBO (Beta-Barium Borate) non-linear crystals cut for type-I phase match with their optical axes orthogonal to each other. Two non-collinear beams of correlated photon pairs at 710 nm are generated by spontaneous-parametric down-conversion (SPDC). A dichroic mirror (D) reflects the pump beam back, while the twin beams are transmitted through a multi-slit placed at the focal plane of *L*_*P*_. They are 100 *μ*m wide, separated by 250 *μ*m intervals. It is shown in ref.^24^ that this arrangement of two crystals generates a polarization-entangled state, and in ref.^23^ it is shown that this configuration with the *L*_*P*_ and the multi-slit generates a spatially entangled state. Therefore, our setup generates photon pairs simultaneously entangled in their polarization and spatial degrees of freedom. This hyperentangled photon source is then used to encode entangled qudits in the transverse positions of the photons, while polarization entanglement is used as an auxiliary resource to implement conditional operations on the qudits. The number of slits sets the dimension of the qudits, which can be changed by displacing vertically a slide containing different multi-slits (see inset in Fig. 1). Therefore, after the multi-slit we can write the state of the photon pairs as

18 $$|{\psi }_{0}\rangle =\frac{|hh\rangle +{e}^{i\lambda }|vv\rangle }{\sqrt{2}}\otimes \frac{1}{\sqrt{d}}\,\sum _{m=1}^{d}\,|m,d-m+1\rangle .$$

**Figure 2:**
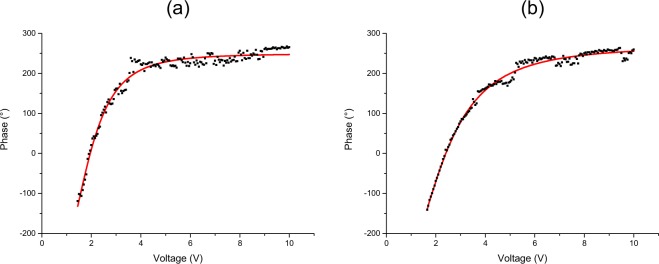
Calibration of the SLM for *d* = 4. The experimental points (black square dots) were fitted using the equation (*A*_1_ − *A*_2_)/[1 + (*x*/*x*_0_)^*p*^] + *A*_2_, and the resulting curve (red line) was used to associate an applied voltage to the corresponding phase introduced. (**a**) calibration for the beam going to detector 1 and (**b**) calibration for the beam going to detector 2. Different calibrations for each photon beam were carried out in order to account for any asymmetry regarding the diffraction in the SLM pixels. In this case, the phases −135°, −67.5°, −22.5°, 0°, 22.5°, 45°, 67.5° and 225° appearing in Eq. (17) were implemented applying, respectively, the voltages 1.350 V, 1.622 V, 1.842 V, 1.952 V, 2.052 V, 2.162 V, 2.272 and 9.900 V in curve (**a**). The values for the curve (**b**) were 1.600 V, 1.932 V, 2.252 V, 2.402 V, 2.552 V, 2.702 V, 2.853 V and 8.45 V.

This corresponds to *α*_*mn*_(0) = *δ*_*m*,*d*−*n*+1_. The conditional operations are applied to the photon pairs by a spatial light modulator (SLM) from the Meadowlark company model D3128. Since the qudits are encoded in the transverse paths of the photon pairs generated by SPDC, these transverse paths must be imaged at the SLM plane such that it can perform the operations $\bar{U}$ and $\bar{V}$ in the form specified in the previous section. For this we use the *L*_*C*_ and *L*_*i*_ lenses to project a magnified image of the multi-slit at the SLM plane (the magnification was necessary to match each slit with a pixel row of the SLM). The SLM modulates only the *h* polarization component, which is an important feature for the conditional operation on the state given by Eq. (18). The SLM screen is split horizontally in two regions - one for each photon (signal and idler) beam - and vertically in *d* regions - one for each slit mode. Different grey scales are used at the SLM screen (see the corresponding inset in Fig. 1) in order to apply the phases *ξ* and *χ* defined in Eqs (15–17). As an example, the SLM calibration curves relating a given voltage (grey scale in the control software) to a certain phase for the case where *d* = 4 is shown on Fig. 2. Although the curves remains the same for the other dimensions, the choice of which voltage corresponds to 0° changed according to the required negative and positive phase range dictated by Eqs (15–17). In this way, we can associate the first term in Eq. (18) with the evolved state *α*(*t*) and the second with the reference state *α*(0). After the SLM, a phase shifter device (Ph-Sh) makes the transformations $|h\rangle \to {e}^{-2i\theta }|v\rangle$ and $|v\rangle \to {e}^{2i\theta }|h\rangle$ on one of the twin photons, resulting in a relative phase 4*θ* between the two terms in Eq. (18).

At this point, the state is given by

19 $$|{\psi }_{1}\rangle =\frac{{e}^{-2i\theta }}{\sqrt{2d}}|hv\rangle \otimes \sum _{m=1}^{d}\,{e}^{i({\xi }_{m}+{\chi }_{d-m+1})}|m,d-m+1\rangle +\frac{{e}^{+2i\theta }}{\sqrt{2d}}|vh\rangle \otimes \sum _{m=1}^{d}\,|m,d-m+1\rangle $$

where we considered the Ph-Sh transformation on the idler polarization state and the quarter-wave plate in the pump path was tilted to make *e*^*iλ*^ = 1. For the interference to be possible we must eliminate the polarization distinguishability. A half-wave plate oriented at 22,5° and a PBS in each arm were used to erase this polarization information. Then, after passing through interference filters (10 nm bandwidth, centered at 710 nm), the photons are collected by microscope lenses *L*_*f*_ and are sent to single photon detectors D1 and D2 through multimode fibers. The multimode fibers are required to couple all the encoded modes of the qudit at the same time. The interference between the initial and the transformed qudit states can be observed through the coincidence counts of the correlated photon pairs, given by Eq. (12), when the Ph-Sh angle *θ* is varied. The fractional topological phases are observed as the phase shifts when maximal visibility is recovered.

The production of hyperentangled photons allows direct measurement of the topological phase without longitudinal path interferometry or post selection procedures. We could observe the interference fringes and attain visibilities above the 50% limit that separates genuine quantum correlations from classically correlated sources. This is the main advantage brought by the present scheme as compared to the previous one published in ref.^21^.

## Experimental Results

**Figure 3:**
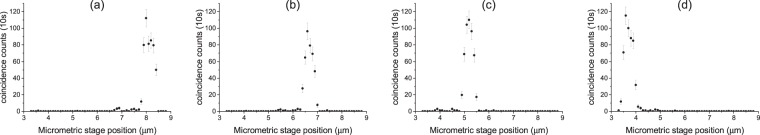
Path correlations for ququarts. The graphs show a single slit scanning in the arm detected by D1 for different positions of another single slit filtering a given slit mode in the arm detected by D2. Namely, the modes coupled to D2 fiber are: (**a**) $|0\rangle$; (**b**) $|1\rangle$; (**c**) $|2\rangle$; and (**d**) $|3\rangle$. The mode $|0\rangle$ is coupled to D1 fiber when we have the lower values in the micrometric stage position, while the mode $|3\rangle$ coupling happens to the larger values. Hence, we only have appreciable coincidence counts when the photon pair is detected in symmetric opposite modes, as expected.

Before performing the measurements, we tested for the state path correlations. This can be done scanning a single slit transversely in each arm on the plane right after the SLM screen, where the multi-slit image is projected. In this way, only one slit mode of each twin photon is coupled to the fiber at a time, and the coincidence counts are recorded. It is worth to mention that the SLM and the Ph-Sh are set to carry out no operations. The results for ququarts are shown in Fig. 3.

Approximating the generated state by a pure state, the area below a certain interval of the curves corresponding to a given slit is related to one coefficient *α*_*mn*_. Proceeding in an analogous way for qubits and qutrits, we have then determined these areas by using a computer software and, after normalization, obtained the following coefficient matrices

$$\begin{array}{c}{\alpha }^{(d=2)}=(\begin{array}{cc}0.006\pm 0.002 & 0.54\pm 0.03\\ 0.45\pm 0.02 & 0.004\pm 0.002\end{array})\\ {\alpha }^{(d=3)}=(\begin{array}{ccc}0.002\pm 0.001 & 0.011\pm 0.005 & 0.36\pm 0.02\\ 0.007\pm 0.002 & 0.37\pm 0.02 & 0.008\pm 0.002\\ 0.28\pm 0.02 & 0.001\pm 0.001 & 0.0007\pm 0.0007\end{array})\\ {\alpha }^{(d=4)}=(\begin{array}{cccc}0.0015\pm 0.0009 & 0.0015\pm 0.0009 & 0.008\pm 0.002 & 0.26\pm 0.01\\ 0.0010\pm 0.0008 & 0.005\pm 0.002 & 0.25\pm 0.01 & 0.006\pm 0.002\\ 0.008\pm 0.002 & 0.20\pm 0.01 & 0.0010\pm 0.0007 & 0.0005\pm 0.0005\\ 0.26\pm 0.01 & 0.0007\pm 0.0006 & 0\pm 0 & 0.0015\pm 0.0009\end{array})\end{array}$$

With these matrices we can calculate the fidelity *F* of the states through the expression $F=|\langle {\phi }_{t}|{\phi }_{e}\rangle {|}^{2}$ where $|{\phi }_{t}\rangle$ is given by Eq. (14) and $|{\phi }_{e}\rangle$ is the state generated experimentally. The results are^:^ *F*^(*d*=2)^ = 0.99 ± 0.04^,^ *F*^(*d*=3)^ = 0.97 ± 0.03 an^*d*^ *F*^(*d*=4)^ = 0.96 ± 0.02.

**Figure 4:**
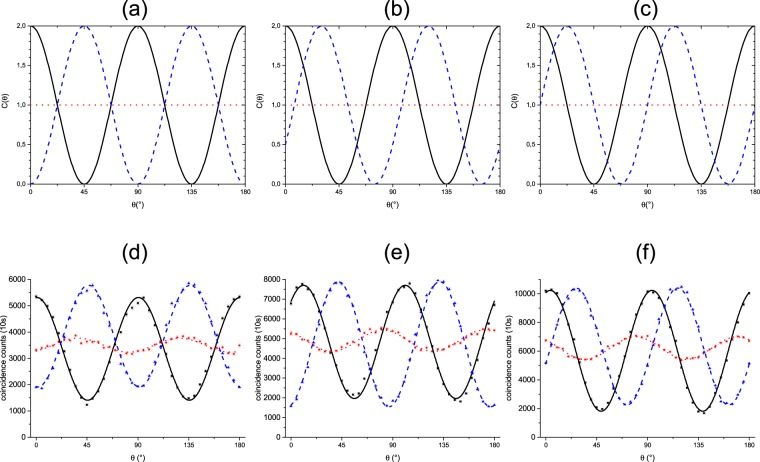
Theoretical quantum interference (**a**–**c**) and fitted experimental coincidence rates (**d**–**f**) are plotted in terms of *θ*. The theoretical curves were generated by using Eqs (12) and (13). The scan angle *θ* is the relative phase between the horizontal and vertical polarization components of one of the twin photons, introduced in the interferometer by a variable phase shifter (Fig. 1). The expected and measured interference patterns of the photon pairs prepared as two qubits (*d* = 2) in path variables under the transformation given by Eq. (15) are shown in (**a** and **d**), as two qutrits (*d* = 3) under the transformation given by Eq. (16) are shown in (**b** and **e**), and as two ququarts (*d* = 4) under the transformation given by Eq. (17) are shown in (**c** and **f**). Black squares and lines correspond to *t* = 0, red circles and dotted lines to *t* = 0.5 and blue triangles and dashed lines to *t* = 1. Zero visibility at *t* = 0.5 in the theoretical curves means that the evolved state at this instant is orthogonal to the initial prepared state. The important aspects to be observed in the interference patterns shown here are the displacement of the patterns obtained at *t* = 1 in relation to the patterns obtained with t = 0. This relative displacement is equal to the fractional topological phase for the two qudit states. At *t* = 1, the SU(*d*) cyclic operation is completed and the evolved state has a topological phase that can be observed from its interference with the initial state, resulting in a relative pattern displacement. The experimental data are corrected only for accidental coincidences, no other noise filters were applied.

Next, we programmed the SLM to implement the operations shown in Eqs (15–17), testing a qubit (*d* = 2), a qutrit (*d* = 3) and a ququart (*d* = 4). The experimental results are shown in Fig. 4.

The expression used for fitting the data was *C* = *A*[1 + *v* cos(4*θ* − *δ*)], where *A*, *v*, and *δ* are fit parameters whose values are shown in Table 1.

**Table 1:** Values of the fit parameters for the experimental data in Fig. 4.

	*t* = 0	*t* = 0.5	*t* = 1
Parameters	*d* = 2		
*A*	(3360 ± 24)	(3500 ± 14)	(3846 ± 24)
*v*	(0.581 ± 0.009)	(0.076 ± 0.006)	(0.502 ± 0.008)
*δ* (°)	(2 ± 1)	(148 ± 4)	(185 ± 1)
	*d* = 3		
*A*	(4832 ± 37)	(4893 ± 17)	(4714 ± 27)
*v*	(0.59 ± 0.01)	(0.110 ± 0.005)	(0.673 ± 0.006)
*δ* (°)	(44 ± 1)	−(35 ± 3)	(166.3 ± 0.6)
	*d* = 4		
*A*	(6026 ± 36)	(6214 ± 17)	(6323 ± 26)
*v*	(0.698 ± 0.007)	(0.126 ± 0.004)	(0.644 ± 0.005)
*δ* (°)	(15.7 ± 0.7)	−(42 ± 2)	(107.2 ± 0.5)

From them we calculate the phase shifts |*δ*(*t* = 1) − *δ*(*t* = 0)| corresponding to the topological phases. We obtained (183 ± 1)°, (122 ± 1)° and (91.5 ± 0.8)° for the qubits, the qutrits and the ququarts, respectively. The uncertainties were obtained making $\sqrt{{({\rm{\Delta }}\delta (t=1))}^{2}-{({\rm{\Delta }}\delta (t=0))}^{2}}$, where Δ*δ* is obtained through the fit made by a computer software. The errors from the fit, in its turn, are affected by the error bars in the in the plots, given by the square root of the coincidence counts following the Poissonian statistics. All measured topological phases in Fig. 4 are in good agreement with the expected theoretical value *γ*_*top*_ = 2*π*/*d*.

In all cases we see that the visibility drops to near zero when *t* = 0.5 and it reaches a value near to the initial one when *t* = 1, as expected from the theory. The small oscillation for *t* = 0.5 is justified by the fact that there is an uncertainty in the phases implemented by the gray scales in the SLM, so that the unitary operations does not correspond perfectly to *t* = 0.5. In this setup the main limiting factor reducing the visibilties is the amount of polarization-entanglement, which was verified through a Bell test. This was done by setting the SLM and the Ph-Sh to zero, so that no transformations were implemented by them. We then followed the method described in ref.^25^ using the HWP1 and HWP2. The visibilities of the curves measured varying the HWP1 for the HWP2 fixed in 0° and 45° were (92.3 ± 0.8)% and (87.5 ± 0.8)% respectively, while the visibilities for the HWP2 fixed in 22.5° and −22.5° were (68.6 ± 0.7)% and (68.9 ± 0.5)% respectively. These values were obtained through a fit made by a computer software. We see that these last two visibilities are compatible with the visibilities for the interference curves in the second row of Fig. 4, which are shown in Table 1. Therefore, we achieved values for the visibilities beyond the 50% limit allowed by classically correlated sources. Another important issue is the improvement of the signal to noise ratio with respect to the measurements in ref.^21^. We can estimate this ratio taking *C*/Δ*C* for a typical coincidence count, lets say approximately the maximum in Fig. 4(f), which gives us 10000/100 = 100. In the previous scheme we have for the ququart normalized coincidence counts approximately 1/0.03 ≈ 33. Then we have a improvement by a factor of ≈3. The real improvement is even larger if we take into account that the acquisition time here is 10 s, while in the other experiment it was 120 s due to the several losses present in the longitudinal interferometer.

## Conclusion

We have measured the fractional topological phases acquired by maximally entangled qudits following cyclic evolutions under local SU(*d*) operations. We set up a quantum optical experiment based on a hyperentangled photon source which is used as a resource to avoid the necessity of longitudinal interferometers or post selection procedures. We obtained experimental results in good agreement with the theoretical prediction for qubits (*d* = 2), qutrits (*d* = 3) and ququarts (*d* = 4). The fractional topological phases could be measured with reasonable signal to noise ratios and the visibilities obtained for the measured interference curves were higher than the limit allowed by classically correlated sources. This is what one would expect once the fringes result from the interference of quantum correlated photons.

In this work, entanglement and conditional local unitary operations in an auxiliary degree of freedom made the global interferometry in longitudinal variables unnecessary for the measurement of the fractional topological phase. Entanglement was an essential resource for measuring these phases just as longitudinal interferometry has been in previous phase measurement experiments. This simplifies the setup and increases the signal to noise ratio. This strategy can be used in different applications starting by increasing the Hilbert space and preparing the particles state in an entangled state in an auxiliary degree of freedom. This scheme offers an interesting perspective for the efficient implementation of quantum phase gates based on topological phases with hyperentangled photon sources.

## Data Availability

The datasets generated during and/or analyzed during the current study are available from the corresponding author on reasonable request.

## References

[1] Berry MV (1984). Quantal phase factors accompanying adiabatic changes. Proc. Roy. Soc. Lond. A.

[2] Aharonov Y, Anandan J (1987). Phase change during a cyclic quantum evolution. Phys. Rev. Lett..

[3] Kwiat PG, Chiao RY (1991). Observation of a nonclassical berry’s phase for the photon. Phys. Rev. Lett..

[4] Brendel J, Dultz W, Martienssen W (1995). Geometric phases in two-photon interference experiments. Phys. Rev. A.

[5] Strekalov DV, Shih YH (1997). Two-photon geometrical phase. Phys. Rev. A.

[6] Sjöqvist E (2000). Geometric phase for entangled spin pairs. Phys. Rev. A.

[7] Hessmo B, Sjöqvist E (2000). Quantal phase for nonmaximally entangled photons. Phys. Rev. A.

[8] Milman P, Mosseri R (2003). Topological phase for entangled two-qubit states. Phys. Rev. Lett..

[9] Milman P (2006). Phase dynamics of entangled qubits. Phys. Rev. A.

[10] Tomita A, Chiao RY (1986). Observation of berry’s topological phase by use of an optical fiber. Phys. Rev. Lett..

[11] Bitter T, Dubbers D (1987). Manifestation of berry’s topological phase in neutron spin rotation. Phys. Rev. Lett..

[12] Richardson DJ, Kilvington AI, Green K, Lamoreaux SK (1988). Demonstration of berry’s phase using stored ultracold neutrons. Phys. Rev. Lett..

[13] Souza CER, Huguenin JAO, Milman P, Khoury AZ (2007). Topological phase for spin-orbit transformations on a laser beam. Phys. Rev. Lett..

[14] Du J, Zhu J, Shi M, Peng X, Suter D (2007). Experimental observation of a topological phase in the maximally entangled state of a pair of qubits. Phys. Rev. A.

[15] Oxman LE, Khoury AZ (2011). Fractional topological phase for entangled qudits. Phys. Rev. Lett..

[16] Johansson M, Ericsson M, Singh K, Sjöqvist E, Williamson MS (2012). Topological phases and multiqubit entanglement. Phys. Rev. A.

[17] Khoury AZ, Oxman LE, Marques B, Matoso A, Pádua S (2013). Fractional topological phase on spatially encoded photonic qudits. Phys. Rev. A.

[18] Johansson M, Khoury AZ, Singh K, Sjöqvist E (2013). Three-qubit topological phase on entangled photon pairs. Phys. Rev. A.

[19] Khoury AZ, Oxman LE (2014). Topological phase structure of entangled qudits. Phys. Rev. A.

[20] Loredo JC, Broome MA, Smith DH, White AG (2014). Observation of entanglement-dependent two-particle holonomic phase. Phys. Rev. Lett..

[21] Matoso AA (2016). Experimental observation of fractional topological phases with photonic qudits. Phys. Rev. A.

[22] Hall, B. C. *Lie Groups*, *Lie Algebras*, *and Representations: An Elementary Introduction* (Springer International Publishing, 2015).

[23] Neves L (2005). Generation of entangled states of qudits using twin photons. Phys. Rev. Lett..

[24] Kwiat PG, Waks E, White AG, Appelbaum I, Eberhard PH (1999). Ultrabright source of polarization-entangled photons. Phys. Rev. A.

[25] Kim T, Fiorentino M, Wong FNC (2006). Phase-stable source of polarization-entangled photons using a polarization sagnac interferometer. Phys. Rev. A.

